# Insights into morphometry and topography of corpus callosum among Indians

**DOI:** 10.6026/973206300191063

**Published:** 2023-11-30

**Authors:** Varalakshmi KL, SR Anusha, Prajwal K Muthaiah, Harshini Jain BS

**Affiliations:** 1 Department of Anatomy, MVJ Medical College and Research Hospital, Hoskote, Bangalore; India

**Keywords:** Corpus callosum, anterior commissure, lamina terminalis, Alzheimer's disease

## Abstract

Corpus callosum is one of the major association fibre of brain performs an integral role of integration and communication of information between the two hemispheres. 50 formalin fixed cerebral hemispheres (25 right and 25 left) were used for the study. The longitudinal and vertical length of brain, longitudinal length and height of corpus callosum, distance of corpus callosum from various landmarks such as frontal and occipital pole, anterior commissure, lamina terminalis, and highest point on parietal pole and width of different parts of corpus callosum and height were measured. Results were analysed statistically. The results showed positive correlation between the longitudinal dimension of brain and all other parameters. Morphometric variation in size and its relation to nearby structures are seen in many neurological and psychiatric conditions such as Alzheimer's disease, Schizophrenia and bipolar disorders. Hence the present study can be used as reference by neurologist, neurosurgeons and psychiatrists.

## Background:

The two cerebral hemispheres are interconnected by thick bundles of commissural fibers known as corpus callosum (CC). The corpus callosum is made up of four parts namely rostrum, genu, trunk and splenium from anterior to posterior. [1] The genu of corpus callosum lays 4 cm posterior to the frontal pole. Rostrum (named so due to its resemblance to bird's beak) is a thin downward and backward prolongation from genu to lamina terminalis. Body is the main part of corpus callosum lies between genu and splenium. The splenium lies 6cm anterior to occipital pole. [2] The major function of the corpus callosum is transferring sensory, motor and cognitive information from one hemisphere to other. [3] Visual, auditory and somatosensory information are conveyed by posterior part of corpus callosum. This unique property of transfer of information between the hemisphere is also attributed to 'spread of seizures 'between the two .Hence , corpus callosotomy(callosal sectioning) is considered to be an effective method of reducing seizure attacks in some cases of generalized seizures. [4] The first morphometric study of the corpus callosum was conducted by R B Beam in 1906 and concluded that exceptional size of the corpus callosum is responsible for extra ordinary intellectual activities, hence the size of corpus callosum directly proportional to intellectual activity of an individual. [5] Corpus callosal malformations are associated with many neurological disorders like schizophrenia, spastic cerebral palsy, hypoxic ischemic encephalopathy and also lead to dyslexia, aphasia and learning disabilities. The Length and total area of corpus callosum is reduced in patients with schizophrenia.[6] In autism fractional anisotropy of corpus callosum is remarkably lessened and there is impaired connectivity between corpus callosum and other major tracts [7]. Therefore, it is of interest to understand the morphometric measurement of corpus callosum as well as it is topographical relations with other nearby anatomical structures of the brain.

## Materials and Method:

The present study is conducted on 50 cerebral hemispheres (25 right and 25 left) obtained from the department of anatomy MVJ medical College and Research hospital. The formalin fixed brain specimens were carefully sectioned in the mid sagittal plane passing through the septum pellucidum using brain knife. The brain specimens with gross anatomical abnormalities, pathological and intracranial lesions were excluded from the study. The following parameters were measured.

[1] Distance between the frontal pole to occipital pole of brain-Longitudinal dimensions of brain-XY

[2] Vertical height of brain along the line passing from central sulcus on the superomedial surface of brain intersecting midpoint of corpus callosum and ending in inferomedial border-AB

[3] Longitudinal dimension of corpus callosum/distance between genu to splenium of corpus callosum-DE

[4] Distance between frontal pole of the brain to genu of corpus callosum -DF

[5] Distance between the occipital pole of the brain to splenium-EG

[6] Distance between genu of corpus callosum to anterior commissure-DH

[7] Distance between genu of corpus callosum to anterior end of lamina terminalis-DI

[8] Height of corpus callosum: To measure the height three points were taken. First point at lowermost point of genu (Point 1), second point at lowermost point of splenium (Point 2). Third point is at highest point on the upper border of body of corpus callosum. Draw a horizontal line between point 1 and point 2 (Line 1). Draw a second line parallel to line 1 and calculate the distance between the two lines which corresponds to the height of corpus callosum-MN

[9] Distance between the corpus callosum to highest point on parietal lobe - PQ

[10] Width of corpus callosum: Width of corpus callosum was measured in following regions

1) Maximum width of rostrum

2) Maximum width of genu

3) Maximum width of body

4) Maximum width of splenium

All these parameters were measured with the help of digital vernier caliper with 0.01mm accuracy. The measurements were performed twice by two observers to avoid inter observer difference.

## Statistics:

Statistical analysis was performed by using SPSS 22. The mean and standard deviation was calculated for all the parameters. The means of different parameters were compared using Student t test. The correlation between the dimensions of brain and corpus callosum was measured using Pearson Coefficient. P value of less than 0.05 was considered significant.

## Results:

50 cerebral hemispheres (25 right and 25 left) obtained for dissection purpose is used for the study. The mean value of longitudinal dimension of brain extending from frontal pole to occipital pole was 15.32 ± 0.866cm.The mean value of vertical dimension of brain was 9.1 ± 0.77 cm .The mean value of longitudinal dimension of corpus callosum was 6.85 ± 0.47cm.The mean value of distance between the frontal pole to anterior most point of corpus callosum(genu) was 5.4 ±0.57 cm whereas distance between occipital pole to posterior most point of corpus callosum (splenium) was 3.1 ± 0.25 cm. Height of corpus callosum was 2.61 ± 0.37cm . The mean value of distance between the corpus callosum to upper most point of parietal lobe was 3.51 ± 0.36cm. The mean value of distance between the genu of corpus callosum to lamina terminalis and anterior commissure was 2.38 ±0.27 cm and 2.98±0.22 cm respectively. The mean, standard deviation, range of all these parameters were measured for both the sides. The values are depicted in Table 1,Table 2.

Pearson's coefficient was used to measure the correlation between dimensions of brain and corpus callosum. The results showed positive correlation between the longitudinal dimension of brain (XY) and all other parameters. The correlation was strongest with DF (0.64) followed by longitudinal dimension of corpus callosum (DE) which was 0.33. Weak correlation was seen between XY and DH (0.07), XY and PQ (0.01). The vertical length of brain (AB) showed weak positive correlation with all parameters. The longitudinal dimensions of corpus callosum (DE) had strong positive correlation with XY, MN. The distance of genu of corpus callosum to frontal pole showed positive linear correlation with XY and EG. Distance between the genu of corpus callosum to lamina terminalis (DI) showed positive correlation with DH and MN. Distance between the genu of corpus callosum to anterior commissure (DH) had positive linear correlation with DI and MN.

## Discussion:

Corpus callosum is a bundle of nerve fiber which reflects the cognitive status of an individual in various neuropathological conditions and behavioral development [8]. The Corpus callosum develops after birth during the first 1-4 years and development extends up to 30 years [9]. The thickness of corpus callosum is directly proportional to the level of intelligence, problem solving and analysis ability of an individual [10]. It was reported that the thickness of corpus callosum markedly reduced in a patient with subcortical ischemic vascular dementia. In cases of myelin damage such as leukoencephalopathy, myelination disorder, metabolic diseases affecting white matter, hypoxic-ischemic encephalopathy, diffuse axonal injury due to trauma, and hydrocephalus thinning of corpus callosum is observed. Damage to the corpus callosum during development period or callosal agenesis is associated with poor neurological and neuropsychological outcome [11]. Hensel et al. conducted comparative study on corpus callosum of normal healthy individual and Alzheimer's disease which showed significant difference in callosal area showing callosal atrophy in Alzheimer's patients. [12] Morphometric measurements of corpus callosum also vary with age, gender, handedness and size of brain. Few studies also mentioned about the sexual difference in size of corpus callosum suggesting the bulbous growth of splenium in females compared to male [14]. In a study by Yakovlev and Leucours observed tremendous burst in myelination of corpus callosum extends up to 7-10 years [10]. Bishop and Wahlstein conducted study on 19 corpus callosums, suggested that there is no strong evidence to prove the existence of gender-based difference in size and shape of corpus callosum. Same finding was observed in the study by Witelson *et al*. [15].

The current study was conducted on cadaveric brains used mainly for dissection purpose. Hence age and sex of specimens were unknown. In the present study the standard deviations of all parameters were very minimal close to mean suggesting negligible variability between the specimens. The values of present study well correlated with previous studies as shown in Table 3. Positive correlation is found between longitudinal dimensions of brain and all other parameters which is in agreement with the findings of Patra *et al*. [4].

Numerous studies have been conducted on corpus callosum using radiological methods like MRI, Computed tomography, diffusion tensor imaging (DTI), trans fontanelle ultrasonography, obstetric Ultra Sound, transvaginal US, and fetal MRI. Study of corpus callosum in pediatric age group using MRI by Bayar OK *et al*. suggested that thickness of different parts of corpus callosum increases with age irrespective of gender. [16] Kirthika *et al*. conducted retrospective study on fetal corpus callosum using MRI and concluded that normal embryological development of nervous system in fetus can be measured by using correlation between corpus callosum and biparietal diameter. [17] MRI study of corpus callosum in dyslexic children showed decreased size of genu. Rounded corpus callosum and reduced size of rostrum is seen in Down's syndrome[18].

## Conclusion:

Data obtained from the present study can be used as reference values to identify the pathological condition or can be used to prevent the undesirable consequences during neurosurgeries. Variations in morphometric measurements might be proportional to degree of impairment of functional interactions within the hemisphere and with the opposite hemisphere. The results of current study can also use as reference value to compare with other populations and ethnic group.

## Figures and Tables

**Table 1:** Showing the values of different parameters of brain and corpus callosum on right and left cerebral hemispheres

**Parameters In cm**	**RIGHT**				**Left**				
	**Min**	**Max**	**Mean**	**SD**	**Min**	**Max**	**Mean**	**SD**	**P value**
XY	14.3	16.5	15.28	0.76	12.9	16.5	15.36	0.99	0.76
AB	7.93	9.82	9.06	0.51	7.52	11.9	9.62	0.87	0.33
DE	5.95	8.07	6.85	0.52	5.42	7.32	6.85	0.43	0.98
DF	4.74	6.72	5.55	0.54	4.16	6.53	5.26	0.57	0.06
EG	2.71	4.01	3.23	0.28	2.64	3.66	3.09	0.19	0.05*
DH	2.38	3.51	2.992	0.23	2.63	3.49	2.98	0.22	0.07
DI	1.98	3.41	2.48	0.32	1.88	2.63	2.281	0.162	0.95
MN	1.71	3.01	2.49	0.33	2.04	3.52	2.74	0.37	0.01*
PQ	2.54	4.39	2.99	0.23	3.05	4.12	3.48	0.35	0.68

**Table 2:** Width of different parts of corpus callosum

	**Rostrum**	**Genu**	**Body**	**Splenium**
Minimum	2 mm	8.3 mm	4.4mm	7.3mm
Maximum	3.6 mm	13.8mm	8.5mm	15.3mm
Mean	2.79mm	11.15mm	6.5mm	11.08mm
Standard Deviation	0.38mm	1.73mm	1.12mm	1.88mm

**Table 3:** Comparison of morphometric values of present study with similar studies conducted on cadaveric brain.

**Parameters**	**Reference[5]50 Brains**	**Ref.[3]37 brains**	**Ref.[4]50 cerebral hemispheres**	**Present study 50 cerebral hemisphere**
Longitudinal dimensions of brain(XY)	16.53±0.72	15.82±1.02	15.47±0.94	15.32±0.8
Vertical height of brain(AB)		10.24±7.88	9.48±0.83	9.1±0.77
Longitudinal dimension of corpus callosum(DE)	7.20±0.45	7.21±5.67	6.96±0.55	6.85±0.47
Distance between frontal pole to genu (DF)	3.27±0.41	3.606	3.31±0.29	3.1±0.25
Distance between the occipital pole of the brain to splenium-EG	4.17±0.42	6.073		5.65±0.5
Distance between genu of CC to anterior commissure-DH	2.71±0.34	5.4±0.57		2.98±0.22
Distance between genu of CC to anterior end of lamina terminalis-DI	2.87±0.38			2.38±0.27
Height of CC(MN)	2.63±0.48	2.33±2.42		3.51±0.36
